# Patient and Family Member-Led Research in the Intensive Care Unit: A Novel Approach to Patient-Centered Research

**DOI:** 10.1371/journal.pone.0160947

**Published:** 2016-08-05

**Authors:** Marlyn Gill, Sean M. Bagshaw, Emily McKenzie, Peter Oxland, Donna Oswell, Debbie Boulton, Daniel J. Niven, Melissa L. Potestio, Svetlana Shklarov, Nancy Marlett, Henry T. Stelfox, Critical Care Strategic Clinical Network

**Affiliations:** 1 PaCER (Patient and Community Engagement Research) Program, University of Calgary, Calgary, Canada; 2 Alberta Health Services, Alberta, Canada; 3 Division of Critical Care Medicine, University of Alberta, Edmonton, Canada; 4 Department of Community Health Sciences, University of Calgary, Calgary, Canada; 5 Department of Critical Care Medicine, University of Calgary, Calgary, Canada; Yokohama City University, JAPAN

## Abstract

**Introduction:**

Engaging patients and family members as partners in research increases the relevance of study results and enhances patient-centered care; how to best engage patients and families in research is unknown.

**Methods:**

We tested a novel research approach that engages and trains patients and family members as researchers to see if we could understand and describe the experiences of patients admitted to the intensive care unit (ICU) and their families. Former patients and family members conducted focus groups and interviews with patients (n = 11) and families of surviving (n = 14) and deceased (n = 7) patients from 13 ICUs in Alberta Canada, and analyzed data using conventional content analysis. Separate blinded qualitative researchers conducted an independent analysis.

**Results:**

Participants described three phases in the patient/family “ICU journey”; admission to ICU, daily care in ICU, and post-ICU experience. Admission to ICU was characterized by *family shock and disorientation* with families needing the *presence and support of a provider*. Participants described five important elements of daily care: *honoring the patient’s voice*, *the need to know*, *decision-making*, *medical care*, and *culture in ICU*. The post-ICU experience was characterized by the challenges of the *transition from ICU to a hospital ward* and *long-term effects of critical illness*. These “ICU journey” experiences were described as integral to *appropriate interactions with the care team* and *comfort and trust in the ICU*, which were perceived as essential for a *community of caring*. Participants provided suggestions for improvement: 1) *provide a dedicated family navigator*, 2) *increase provider awareness of the fragility of family trust*, 3) *improve provider communication skills*, 4) *improve the transition from ICU to hospital ward*, and 5) *inform patients about the long-term effects of critical illness*. Analyses by independent qualitative researchers identified similar themes.

**Conclusions:**

Patient and family member-led research is feasible and can identify opportunities for improving care.

## Introduction

Actively engaging stakeholders (e.g., patients and family members) from research inception to implementation is increasingly used in health sciences research.[1–4] The rationale for this approach is that outputs are more relevant to stakeholder needs and thereby facilitate implementation of the results, avoid waste in research and enhance patient-centered care.[5–7]

A key challenge has been how to best engage patients and families. Patient engagement has been introduced at different stages of health research and with various levels of participation, but efforts to date are framed and initiated from a provider perspective, may not fully engage patients and families and result in lost opportunities.[8] A recent systematic review[9] found potential benefits to engagement that included facilitating patients’ recognition of their condition and empowering them to optimize management. Challenges included identification of appropriate patients and families for engagement, and concerns that they may feel overburdened and inadequately prepared to participate. This is particularly true for critically ill patients admitted to intensive care units (ICUs), whose severity of illness means that they often do not remember their care experience, have prolonged recovery periods, and whose family members may be too overwhelmed by the circumstances to participate.[10]

We employed a novel research approach that engages and trains patients and family members as researchers to understand and describe the experiences of patients admitted to the ICU and their family members, and to identify opportunities for improvement. We focused on patients with a previous episode of critical illness (i.e., ICU admission) and their family members because previous studies have suggested that they are a challenging population to engage.[10]

## Methods

### Approach

We contracted Patient and Community Engagement Research (PaCER),[11–13] a research team that trains patients and family members of patients as researchers. The program is twelve months in duration and includes both courses (theory of patient engagement research, ethics, qualitative research methods) and a project-based internship. Upon completion of the program participants are expected to be able to understand patient engagement research, appreciate salutogenic approaches[14] to health and have developed the skills to propose and conduct basic patient engagement research studies using field observation, surveys, focus groups and narrative interviews (www.obrieniph.ucalgary.ca/research/programs-units-centres/pacer/training). The PaCER team, comprised of four family members of patients (MG, PO, DO, DB), led and conducted all phases of the research with patients and families. This encompassed refinement of the research question, study design, data collection, analysis and interpretation, and development of a draft manuscript. Members of the research team (SMB, EM, DN, MP, HTS), not part of PaCER, provided resources to support the work, facilitated identification of patients and family members for participation, interpreted the synthesized data, and revised the manuscript for important intellectual content. This approach was designed to explore patient and family experiences and perspectives of ICU care without undue influence of providers or “traditional” researchers.

The research question for the study was: what about healthcare works and what does not work for patients and family members of patients admitted to ICU?

### Sampling and Recruitment

The recruitment strategy was to make information about the research study broadly available and allow individuals to self-identify and contact PaCER. Managers from 14 medical-surgical ICUs in a geographically defined healthcare system serving a population of 4 million residents disseminated information about the project via email, website, telephone calls, or personal contact to patients and families with ICU experience. Eligible participants included individuals 18 years of age and older, English-speaking, patients or family members of patients (surviving or deceased) who had received care in ICU within the last two years.

### Data Collection

PaCER conducted five focus groups (23 unique participants) and eight telephone interviews (9 unique participants) to ensure inclusion of participants from geographically remote communities. Prior to each focus group and interview, participants were contacted by telephone (MG) to explain the study and invite questions. Focus groups were conducted in multi-purpose rooms within healthcare facilities previously confirmed by participants as being comfortable for them. Only participants and facilitators were present and refreshment breaks allowed for socializing. Interviews were scheduled according to participant convenience and conducted while participants were at home. The PaCER “Set-Collect-Reflect” research approach[13] was used to frame the data collection and analysis process. During the initial focus group, participants shared experiences that they wished had been different (i.e., potential improvements) and thereby “Set” the initial direction (i.e., guiding questions, see S1 Appendix) for the research. Next, PaCER conducted three focus groups and eight interviews to “Collect” data on the experiences of the participants with ICU care. During the final “Reflect” focus group, representatives from the four focus groups and interviews were invited to attend a final group to ensure their experiences were accurately reflected in the analyses and to derive working theory and recommendations.[15] Each focus group (facilitated by MG and two other PaCER members) lasted approximately 5 hours, and each interview (interviewer MG) 60 to 90 minutes. Participants were asked to describe their experience in ICU, and prompts were used when necessary to elicit thorough descriptions of what worked and did not work. Focus groups and interviews were audio taped and transcribed. Field notes, including visual observations, were recorded. Documentation and review of flip chart notes during the focus groups allowed for verification of PaCER’s understanding of participant experiences.

### Analysis

Qualitative methods using conventional content analyses[16] were conducted to describe the “lived” experience of individuals. Data was coded manually into emerging sub-themes, as PaCER read and listened to the data repeatedly to achieve immersion.[17] Sub-themes were grouped into larger themes.[18] A final list of themes, sub-themes and the relationship between them was agreed upon through discussion and consensus. Data collection and analysis occurred simultaneously and continued until no further unique themes emerged from successive focus groups and interviews.[17, 19]

PaCER used several strategies to evaluate the credibility and trustworthiness of the data and analysis: 1. Possible biases of PaCER with respect to personal experience were noted and revisited[18]; 2. Substantial time was dedicated to build relationships to allow for uninhibited interaction among participants and easy expression of views[20]; 3. Multiple methods of data collection and data recording were used to enhance contextual validation[15, 21]; 4. A minimum of three unique participant sources were used to classify an experience as a theme[15]; 5. To verify credibility, PaCER conducted analysis debriefing with colleagues, external auditors (SS, NM) and members of the broader research team (HTS, EM, MP)[15, 22]; 6. Member checks, or solicitation of participant feedback, were used at three separate stages. First, flip chart notes were reviewed at the end of each focus group, with participant comments and edits invited. Second, participants in the final focus group reviewed and sorted findings. Third, before finalizing results, copies of the analytic framework were sent to participants for feedback.[15, 21]

To compare the PaCER approach to that of ‘traditional’ researchers, an independent qualitative research team, blind to the PaCER analyses, conducted a content analysis of the audio taped focus group and interview transcripts. Ethics approval was obtained from the University of Calgary (REB13-1157) and University of Alberta (Pro00048227). All participants provided written informed consent to participate in the study.

## Results

**Fig 1:**
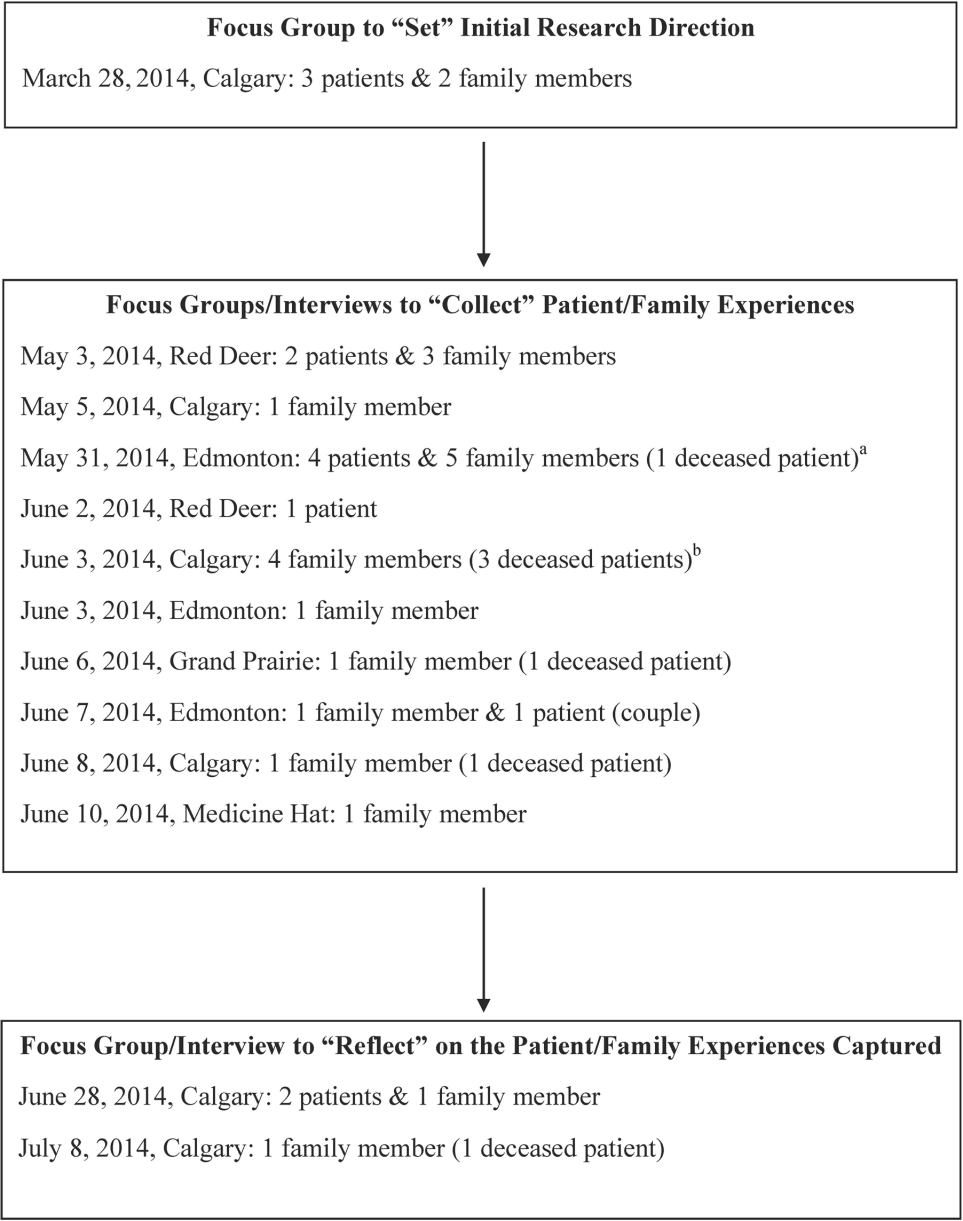
Flow of focus groups and interviews. Focus groups and interviews to sequentially “set” the initial direction of the research, “collect” data on participant experiences with ICU care and “reflect” on the analyses to derive working theory and recommendations. ^a^ Includes one participant from Fort McMurray. ^b^ Includes one participant from Lethbridge.

Thirty-nine individuals contacted PaCER to inquire about participating in the study, of which 32 consented, four were unable to participate due to conflicting commitments and three declined citing ongoing difficulties with memories (Fig 1). Participants had experienced care in 13 adult medical-surgical ICUs across seven cities. The characteristics of the participants are summarized in Table 1.

**Table 1:** Participant Characteristics.

Characteristics		Participants (n = 32)
Sex		
	Male	15
	Female	17
Age, years		
	<50	8
	50–64	15
	65+	9
Patient/Family		
	Patient	11
	Family of surviving patient	14
	Family of deceased patient	7
Experience with more than one episode of critical illness^a^		17
Duration of patient ICU stay		
	1 to 7 days	6
	8 days to 4 weeks	12
	> 4 weeks	14
Duration of patient hospital stay		
	1 to 30 days	12
	31 days to 2 months	11
	> 2 months	9
Type of hospital^b^		
	Tertiary referral hospital	16
	Community, large urban center^c^	14
	Community, small urban center^c^	10
Hospital academic status^b^^,^ ^d^		
	Teaching	23
	Non-teaching	13

^a^ Participants that had more than one experience with critical illness as either a patient or family member of a patient (i.e., experience with more than one admission to an ICU).

^b^ Numbers total to more than 32 as 7 patients and/or family members experienced care in more than one hospital.

^c^ Community hospitals classified according to whether the urban center had more or less than 1 million residents.

^d^ Hospital academic status classified according to whether medical, nursing, respiratory therapy and allied healthcare trainees routinely participate in patient care.

Three major phases in the patient and family ICU “journey” were identified; admission to ICU, daily care in the ICU, and post-ICU experience.

### 1. Admission to ICU

Family members described the experience surrounding admission to ICU. Most patients reported little, if any, memory of this experience. Two themes were identified (Table 2).

**Table 2:** Patient and Family Experiences with Care.

Themes^a^		Exemplar Quote
Admission to ICU		
1. Family shock and disorientation		It took me about three days to get my mind to wrap around the thing. I just kept wondering, how did this happen? (family member of surviving patient)
2. Presence and support of a provider		You know it would really help if there was one person, the same person, [to] explain what is going on…someone who knows the system–who knows how ICU works. (family member of surviving patient)
Daily care in the ICU		
1. Honoring the patient’s voice		
	Patient’s (in)ability to communicate	It’s scary and you finally figure out you can slide your finger out of the sensor and they come in…and your arms are tied down the nurses would just say–“I don’t know what this means,” and they would just turn away and walk off. Like am I supposed to write it with my tongue? I have no idea how to get what I want…like the bathroom (patient)
	Family is the patient’s voice	It’s like, oh my God I am speaking for him, he can’t speak for himself. It’s a huge responsibility … I couldn’t miss a beat … and had to be there, the one talking to the doctors. (family member of surviving patient)
2. The need to know		
	Daily updates	We always knew what was going on … we knew his condition daily … it was always clear as to what they were doing. (family member of deceased patient)
	Timely updates for major changes	I felt like I missed it. I felt like I should have been there … I left the hospital and they didn’t phone me and it was such a major change. (family member of surviving patient)
	Keeping patient information private	Someone, not part of the family would arrive and the nurses would give them information about my husband. This upset us a lot. (family member of surviving patient)
3.Decision-making		
	Discussions of prognosis	Husband: When she got into ICU he [physician] informed me that she probably would have to go to … a nursing home so why not just let [her] go … [she] was in the room and she was awake and she heard it. Wife: I don’t remember it. Husband: … you don’t remember but you heard–you had a shocked look on your face. You couldn’t talk because you had the breathing tube but she had a very shocked look on her face. She understood. She didn’t want to die. (family member and patient conversation)
	Balance of hope and reality	Another excellent thing was they left hope … yet they were realistic. (family member of deceased patient)
	Goals of care	There was nothing signed. It was all verbal. No one had ever said that he was going to be Do Not Resuscitate. (family member of surviving patient)
4. Medical care		
	Providing the best medical care	The level of care my son received … was nothing short of exceptional. (family member of deceased patient)
	Continuity of providers	They were constantly changing … To have someone stable would be nice. You kind of dreaded the shift and doctor change. (family member of deceased patient)
5. Culture in ICU		
	Access to support	I felt like I was imposing on him [social worker] … I was afraid to knock on his door. He was never on the unit. (family member of surviving patient)
	Inviting family to be part of the care team	I wanted to get involved but I guess I was more of a burden to them … I’d ask and I would get the sigh … The reality is … you don’t feel part of the team. There’s just something missing. (family member of surviving patient)
	Allowing family to be with the patient	My brothers and I we slept there every night. (family member of deceased patient)
	ICU facilities for families	Sometimes you want some time by yourself … I went to the bathroom at one point, just to get away. (family member of surviving patient)
Post-ICU Experience		
1. Transition from ICU to a hospital ward		I’m trying to understand the picture of the future and the people in ICU had no idea about rehab. The ability of people to look down the chain would have been helpful. (family member of surviving patient)
2. Long-term effects of critical illness		I still get dizzy spells, memory loss. I forget the rest of the sentence I was going to say. I get time lapses, get chest pains and headaches. I’m not sure what is normal for what I have gone through. (patient)

^a^ Data presented as themes with sub-themes within three phases in the patient and family ICU “journey”; admission to ICU, daily care in the ICU, and post-ICU experience.

#### Family Shock and Disorientation

Family members reported shock and disbelief, followed by disorientation and difficulty in adjusting to invasive medical interventions, equipment, and the “alien” culture of ICU. Some reported that they never adjusted or felt comfortable.

#### Presence and Support of a Provider

Families outlined their need for the continuous presence of a proactive, friendly and informed provider. They wanted clear, consistent and complete information from someone who acted as their “eyes and ears,” because they didn’t always know what to ask.

### 2. Daily Care in the ICU

Participants articulated experiences of daily care in the ICU related to interactions with the care team and efforts to establish comfort and trust in the ICU. They reported these experiences to be integral for a community of caring. Five themes were identified (Table 2).

#### Honoring the Patient’s Voice

Many patients were unable to speak for themselves during their ICU stay (e.g., altered level of consciousness). Family members articulated the stress of the responsibility of being the patient’s spokesperson. They noted that at times they felt heard by providers, while at other times dismissed. Families reported that even as a patient’s clinical condition improved, they frequently acted as interpreters, reading lips or guessing patient needs, because patients found communication challenging (e.g., quiet, hoarse voice) and perceived providers to be impatient (noting sighs, raised eyebrows, “tutting” and other non-verbal signs when they asked questions).

#### The Need to Know

Families reported a need to know the patient’s status and manage dissemination of this information. This provided them with a sense of control. The degree to which families reported being kept informed varied substantially; those who indicated that they were kept well informed reported being more trusting of providers, and comforted that their relative “was in good hands”. Some families found it difficult to leave the hospital because they lacked trust that they would be informed of changes in patient status.

#### Decision-Making

Families’ ability to make decisions about patient care, and have confidence in their decisions, was impacted by the information and support they received. Families who were well informed and supported indicated that they were confident in their decision-making. Others found it difficult to trust providers and often remained unsure of their decisions. The timing, place, and manner of information sharing were important factors in decision-making.

#### Medical Care

Participants reported the quality of medical care was exceptionally high and at times taken for granted. Participants perceived provider turnover to negatively affect the delivery of care, hinder the development of trusting family-provider relationships, which made it more difficult for families to ask questions and receive information.

#### Culture in ICU

It was important for the family to be with the patient, to be invited to be part of the care team, and to have access to support. Families perceived that, unless they repeatedly asked questions or insisted on being present, they were excluded from important information and substantive contact with the patient, which heightened their anxiety. Some participants noted the culture in ICU was not inviting and there was little acknowledgment of the emotional stress families experienced and their need for private “time out” space.[23] Families indicated that being invited to give input and being allowed to help in the care of the patient resulted in less anxious, tense and confrontational relationships with providers and greater satisfaction with the care experience.

### 3. Post-ICU Experience

Participants’ post-ICU experiences were organized into two themes.

#### Transition from ICU to a Hospital Ward

Participants described transitioning from ICU to a hospital ward as challenging. Patients and families reported inadequate preparation to ready them for the move; perceived ICU providers to have little knowledge of what would happen to the patient after they left ICU; and perceived communication between ICU and ward providers to be poor.

#### Long-term Effects of Critical Illness

Participants described anxiety-provoking physical, cognitive and mental health symptoms after returning home, suggestive of post-intensive care syndrome.[24] They were unsure if the symptoms represented long-term consequences of treatments received in ICU, and were hesitant to consult their primary care physicians, who they perceived to have inadequate knowledge of, and experience with, long-term sequelae of critical illness.

### Suggestions for Improvement

Participants provided five suggestions for improvement (Table 3).

**Table 3:** Patient and Family Suggestions for Improvement.

Suggestion	Exemplar Quote
1. Provide a dedicated family navigator	It would have been great to have a person who knew how ICU works to help me understand… a kind of guide to things related to ICU. (family member of surviving patient)
2. Increase provider awareness of the fragility of family trust	We camped out for nine days–we took over the waiting room … We had no trust. (family member of surviving patient)
3. Improve provider communication skills	Anyone who had anything to do with that particular nurse noted that she was not sensitive, she did not communicate well and that threw everyone off. (family member of deceased patient)
4. Improve the transition from ICU to a hospital ward	It was a total culture shock…the transition was bad … Are they up-to-date with her case? (family member of deceased patient)
5. Inform patients about the long-term effects of critical illness	Not enough information was provided to us to help me know what to expect… Be prepared for what might happen to you. (patient)

1. **Provide a Dedicated Family Navigator.** Family members recommended that ICUs employ dedicated providers to liaise with families. These individuals would have strong communication skills, be empathetic, have ICU experience (e.g., existing members of the ICU such as ward clerks, social workers, volunteers etc.), and be available throughout a patient’s stay as needed.
2. **Increase Provider Awareness of the Fragility of Family Trust.** Participants indicated that providers should be informed of the fragility of family trust. Caring providers can be overshadowed by the few who are brusque, inappropriate or leave families feeling a nuisance, vulnerable, fearful and unwelcomed.[25]
3. **Improve Provider Communication Skills.** Participants reported that communication was a key to a patient and family-centered ICU. This included the mode, tone and content, as well as a bidirectional process with their input valued.
4. **Improve the Transition from ICU to a Hospital Ward.** Families recognized the high-risk nature of the transition from ICU to a hospital ward and suggested strategies to improve it. They recommended a joint meeting of the patient, family and the discharging ICU, and admitting ward providers prior to the transition of care to facilitate a comprehensive and shared understanding of the patient’s situation and treatment plan.
5. **Inform Patients about the Long-term Effects of Critical Illness.** Participants requested that patients and families be provided with a summary of medications administered, common long-term effects of critical illness, and contact information for providers with experience in post-intensive care syndrome. They reported this would help them determine what to expect and when to seek assistance.

### Independent Analyses by Qualitative Researchers

A team of three independent qualitative researchers (all with Master’s degrees and 5–10 years of qualitative research experience) blinded to the PaCER analyses identified similar themes and suggestions for improvement with a few notable exceptions (Table 4).[26] They categorized the data into three groupings of content; 1) patient and family experience (5 themes), 2) patient care (6 themes), and 3) patient and family support and resources (5 themes), in contrast to PaCER’s organization of the data into a temporal journey. Additional themes identified included *patient safety and comfort* and *staying connected with friends and family*. The researchers identified additional suggestions for improvement that included *making ICU rules and procedures more readily available* and *providing communication aids for patients who cannot speak*.

**Table 4:** Comparison of PaCER and traditional researcher analyses^a^.

Themes, Subthemes^b^	
PaCER	Traditional Researchers
Admission to ICU	Patient and Family Experience
• Family shock and disorientation	• ICU atmosphere and living
• Presence and support of a provider	• Practical and physical accommodations
Daily care in the ICU	• Patient and family demographics
• Honoring the patient’s voice	• Life post-ICU
ⵔ Patient’s (in)ability to communicate	ⵔ Transition
ⵔ Family is the patient’s voice	ⵔ Discharge preparation
• The need to know	ⵔ Life at home
ⵔ Daily updates	• Coping with illness & impact on Families
ⵔ Timely updates for major changes	Patient Care
ⵔ Keeping patient information private	• Appropriate care
• Decision-making	ⵔ Quality of care
ⵔ Discussions of prognosis	ⵔ Staff knowledge and competency
ⵔ Balance of hope and reality	ⵔ Access to allied health providers
ⵔ Goals of care	ⵔ Trust
• Medical care	• Patient privacy, respect, and dignity
ⵔ Providing the best medical care	• Patient safety and comfort
ⵔ Continuity of providers	• Continuity of care
• Culture in ICU	• Communication
ⵔ Access to support	ⵔ Patient’s ability to communicate
ⵔ Inviting family to be part of the care team	ⵔ Patient & family’s communication with providers
ⵔ Allowing family to be with the patient	ⵔ Communication among providers
ⵔ ICU facilities for families	• Managing expectations
Post-ICU experience	Patient and Family Support & Resources
• Transition from ICU to a Hospital Ward	• Accommodations—emotional support
• Long-term Effects of Critical Illness	• Bedside manner
	• Patient & family involvement in care & decision-making
	• Resources & information given to patients & families
	• Staying connected with friends & family
Suggestions for Improvement	Suggestions for Improvement
• Provide a dedicated family navigator	• Provide a family guide
• Increase provider awareness of the fragility of family trust	• Make ICU rules & procedures more readily available
• Improve provider communication skills	• Provide communication aids for patients who can’t speak
• Improve the transition from ICU to a hospital ward	• Improve transition out of the ICU
• Inform patients about the long-term effects of critical illness	• Inform patients what to expect when they return home
	• Make it easier and more affordable to park
	• Provide access to food/drink for families in the ICU
	• Provide space for families to rest & nap

^a^ Text highlighted in grey represent themes/suggestions for improvement reported in only one set of analyses.

^b^ Data presented according to groupings of content (underlined headings) with nested themes (closed bullets) and subthemes (open bullets).

## Discussion

We employed a novel research approach that engages patients and family members as researchers to explore patient and family care experiences and identify opportunities for improvement. Participants described shared key experiences in the patient and family "ICU journey” related to admission to ICU, daily care in the ICU and the post-ICU experience. Five suggestions for improving the patient and family experience were proposed. Analyses by independent qualitative researchers identified similar themes and suggestions for improvement, but through more of a health system than patient and family lens.

### Patient and Family Role in Research

Traditionally, research studies have been conceptualized, undertaken, and analyzed by health researchers. Patient and family participation has been largely limited to being study subjects.[27–33] Our study demonstrates that patients and families can, and should, play an active role in research. The James Lind Alliance has established an approach for bringing patients, families and providers together to identify and prioritize unanswered questions.[34] The Patient Centered Outcomes Research Institute has placed patients and families at the center of the research enterprise.[35] The research approach employed in this study adds to these initiatives by demonstrating the feasibility and value of not only having patients and/or family members integrated into planning research, but also leading all aspects of the process.[11] The feasibility of the PaCER approach is illustrated by the recruitment of patients and family members to become researchers, their successful completion of a training program and their effective execution of a research project in a timely manner (6 months). Moreover if this approach can be successfully employed with patients who have experienced critical illness and their family members, it should be feasible with less vulnerable populations.[10]

Patient and family member-led research is valuable because it reduces the potential for power relationships that may exist in more traditional researcher-participant scenarios. This thereby lessens the need for reflexivity, the process of critical self-evaluation of researcher’s position and acknowledgment that it may impact both the research process and the outcome.[36] While PaCER and the independent qualitative research team identified broadly similar themes; there were distinctions that reflect important differences in the two research approaches. For example, while the independent qualitative researchers identified improved *access to parking*, *food/drink and spaces for resting* as opportunities to improve care, PaCER identified *increasing provider awareness of the fragility of trust*. More importantly the two groups categorized the data very differently. The independent qualitative research team grouped the content into distinct domains (patient and family experience, patient care, patient and family support and resources), while PaCER organized the data into a temporal patient care journey. This may reflect differences in the two groups’ perspectives (patient and family vs. researcher) and highlights how the lens through which researchers interpret data impacts the results.

Engaging patients and family members throughout the research process holds the promise of addressing the challenges most important to them in a more direct and effective fashion. This could potentially span the spectrum of clinical, health services and population health research including the identification and prioritization of research priorities, knowledge generation, knowledge synthesis and implementation of research findings into practice. While training patients and family members in research methodology is a major investment requiring a long-term commitment to patient and family engagement, our experience suggests it is feasible, will improve the value of research and lead to enhanced patient and family-centered care.[37]

### Lessons for the Care of the Critically Ill

**Fig 2:**
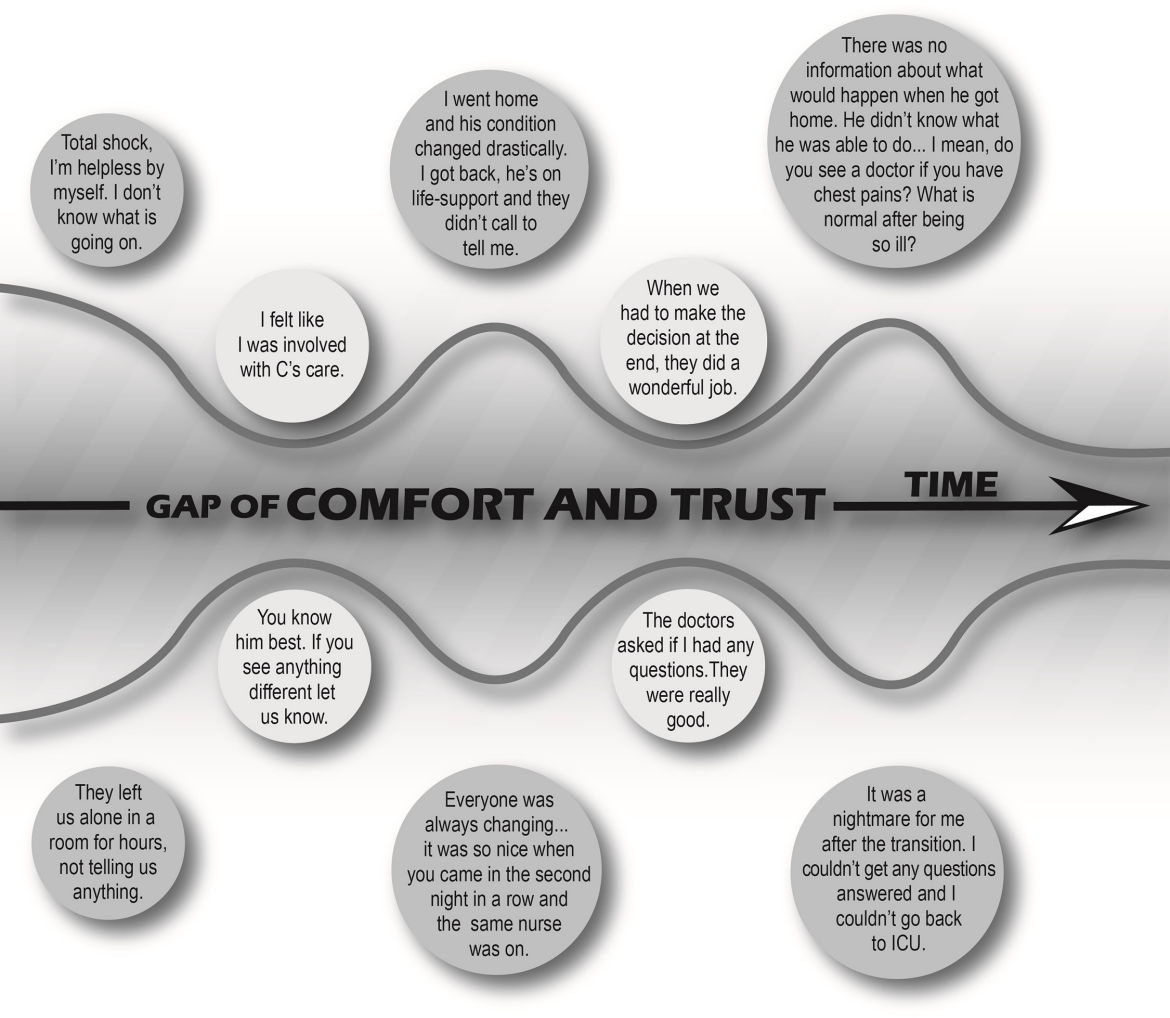
Patient and family comfort and trust in the ICU. Patient and family member interactions with the care team determine comfort and trust in the ICU.

Our study illustrates the type of patient and family experiences that can be described and the lessons learned by patient and family member-led research. Patients and families across 13 institutions from seven cities described common experiences with care, and provided common suggestions for improving care. Furthermore, our results are consistent with observations from other studies that have elicited the patient and family experience with receiving ICU care. For example, Azoulay et al. reported the importance of providing families with consistent information in a survey conducted in ICUs in France,[33] while Nelson et al. described the importance of effective communication and shared-decision-making by focus group participants in the United States.[32] Our data in the context of the pre-existing literature suggest that critically ill patients and their families share common experiences and that initiatives to improve care can target these issues. Key findings include the need for; 1. A culture that integrates patients and families as members of the care team; 2. Support for families during disorienting and distressing times; 3. Effective and consistent communication that allows patients and families to be informed and heard; 4. Minimizing the “revolving door” of providers and the impact of transitions of care; and, 5. Recognition that families are the voice for patients who cannot communicate. Participants identified these as essential for promoting comfort with, and trust in, ICU care settings (Fig 2). It is not just the “what” an ICU offers, but also “how” it is offered, that is important.

For most critically ill patients, the therapeutic alliance is between providers and the family. In our study, families clearly described how to foster that relationship; recognize their stress and disorientation when the patient is admitted, proactively orient them, invite them to be active members of the care team (i.e., attending rounds, helping at the bedside as appropriate) and engage them in bidirectional communication and decision-making. A family guide was suggested as one strategy to help families navigate the ICU experience. Patients and families recognized the stress and risk associated with transitions of care, which can be managed by both minimizing the number of transitions (e.g., same nurse for the same patient) and the impact of those transitions by standardizing handover and engaging patients and family members as agents of continuity (e.g., empower patients and families to ensure consistent approaches to care). Finally, patients’ and families’ experiences with ICU care did not end with discharge, but included both the transition from the ICU to a hospital ward and concerns about the long-term effects of critical illness. For most patients, critical illness is not a discrete and isolated event restricted to the ICU, but part of a longitudinal health journey (even for newly diagnosed conditions and injuries). Patients and families want to understand the long-term ramifications of their illnesses and to be empowered to manage them across the continuum of care. They perceive ICU providers to be better positioned to provide this information than hospital ward providers or family physicians and that strategies to mitigate and manage post-intensive care syndrome should begin in the ICU.

### Limitations

Application of the findings may be limited by transferability to other settings. Our study reports experiences of patients and families cared for within a single geographically defined healthcare system. Experiences may differ in other settings, depending on the way that services are organized and delivered. Patients and families of different ethnic, cultural and socioeconomic backgrounds may have different experiences. However, qualitative research was undertaken to reveal detailed information about experiences, and rigorous methods were used. While the sample size for our study is small (n = 32), we sampled participants from 13 of the 14 adult medical-surgical ICUs within a healthcare system, including all ICUs located outside of the two largest urban centers. Similar themes were expressed during all focus groups and interviews, and trends by type of ICU or participant characteristics were not identified, suggesting that data saturation was achieved and a comprehensive representation of experiences was obtained. Analyses by independent blinded qualitative researchers produced similar results. Research is needed in other jurisdictions, and in other patient and family populations, to confirm these findings, although the feasibility and value of engaging former patients and family members as researchers is likely to be similar.

## Conclusions

In summary, patient and family member-led research is a novel research approach that is feasible to conduct and can be used to identify opportunities for improving care. Study participants described shared key experiences in the patient and family "ICU journey” related to admission to ICU, daily care in the ICU and the post-ICU experience. Five suggestions for improving the patient and family experience were proposed. Patient and family member-led research provides a novel approach for engaging patients and family members and could serve as a valuable extension of conventional health services research approaches.

## Supporting Information

S1 Appendix PaCER: Collect Focus Group. Copy of the focus group guide.
(DOCX)
