# Supplementary material for: Patient and Family Member-Led Research in the Intensive Care Unit: A Novel Approach to Patient-Centered Research
Source: PLoS One. 2016 Aug 5;11(8):e0160947. doi: 10.1371/journal.pone.0160947 (PMC4975402; doi:10.1371/journal.pone.0160947)
Supplement: S1 Appendix — Copy of the focus group guide. (DOCX) [file pone.0160947.s001.docx]

**Appendix 1**

**PaCER: COLLECT Focus Group**

**Exploring patient and family experiences in the daily care of critically ill patients in ICU**

**A. Guiding questions (family)**

•      Tell us about your experiences in ICU that made it possible for you as a family member to feel involved in the daily care of the patient.

 •      Were you kept updated on any changes in your family member’s health when you were not at hospital or asked to leave his/her bedside?  If so, how was this done?

 •      Were you given enough information on the daily routine in ICU or were you sometimes asked to leave without knowing why? Anything that could have been better or different?

 •      Did you feel part of the “care team” or did you sometimes feel “in the way”? If so how was that done? If not, what would you have liked to have happen differently?

 •      Is there anything that you would like to see done differently that would have been more helpful for you?

**B. Guiding questions (patient)**

 •      How did you communicate your needs to staff members?

 •      Were you given information about what to expect when you got home? If so, was it useful?
